# Gastric cancer peritoneal metastases as an immune compartment: immune evasion, systemic immunotherapy and regional treatment strategies

**DOI:** 10.3389/fonc.2026.1980560

**Published:** 2026-10-15

**Authors:** Eoghan Burke

**Affiliations:** Department of Upper Gastrointestinal Surgery, Beaumont Hospital, Dublin, Ireland

**Keywords:** clinical-trial design, CRS–HIPEC, gastric cancer, immune evasion, immunotherapy, intraperitoneal chemotherapy, peritoneal metastases, tumour microenvironment

## Abstract

Gastric cancer peritoneal metastasis (GCPM), particularly associated with diffuse and poorly cohesive cancers, in which tumour cells grow singly or in small, loosely associated groups, presents an anatomical and immunological treatment challenge. This review examines the peritoneal surface and surrounding fluid as an interacting immune compartment comprising tumour implants, malignant ascites, free tumour cells, stroma and immune populations. Human studies identify cytotoxic-cell depletion or exhaustion, macrophage-rich ascites and suppressive soluble mediators; experimental findings support complement-pathway involvement in immune-checkpoint ligand expression. These observations offer a biological explanation for impaired local immunity but do not establish clinical resistance to checkpoint inhibition. Systemic therapy remains the treatment backbone, yet pivotal trials rarely report the Peritoneal Cancer Index (PCI), cytology, ascites response or peritoneal-specific progression. Early studies combine checkpoint blockade with pressurised intraperitoneal aerosol chemotherapy (PIPAC), repeated intraperitoneal paclitaxel or hyperthermic intraperitoneal chemotherapy (HIPEC); direct intraperitoneal antibodies, cytokines and cellular therapies are also under investigation. Feasibility and local pharmacodynamic changes do not establish synergistic benefit. Future trials should compare an added intraperitoneal treatment against an otherwise identical systemic regimen, collect matched blood, ascites and peritoneal tissue samples over time, and relate immune changes to cytological clearance, PCI, symptoms and survival. Operative consolidation requires a separate randomised assessment in prospectively defined responders. Treatment continuity, quality of life, nutrition and hospital-free survival are essential measures of net benefit.

## Introduction

Gastric cancer remains a major global cause of cancer mortality. Demographic modelling estimated approximately 1.1 million new cases and 770,000 deaths in 2020 and projected that, if age-specific rates remain stable, annual cases could approach 1.8 million and deaths 1.3 million by 2040, largely because of population growth and ageing (1). These projections concern absolute numbers of cases and deaths, rather than a universal increase in age-standardised incidence rates. Nevertheless, they mean that health systems will encounter increasing absolute numbers of patients with advanced disease.

Peritoneal metastasis (PM) is central to that burden. A systematic review reported synchronous PM in approximately 10–21% of patients, with higher detection when staging laparoscopy was incorporated (2). In a contemporary nationwide cohort, 23% had synchronous PM and the three-year cumulative incidence of metachronous PM after potentially curative treatment was 22.8%; median survival after synchronous or metachronous PM remained only 3.2 and 2.3 months, respectively, across the whole population (3). Peritoneal recurrence is also a major mode of failure after resection, particularly in diffuse, poorly cohesive and serosa-invasive cancers.

The Western clinical burden may become more visible for two linked reasons. First, the absolute number of gastric cancers is expected to increase with population ageing. Second, several Western registries have described a relative shift towards diffuse or poorly cohesive phenotypes and disease in younger adults (4). A recent Irish national cohort also showed that diffuse-type and signet-ring-cell cancers were disproportionately diagnosed with stage IV disease including peritoneal metastases (5). These observations do not establish that the population incidence of PM is rising in every Western country. They do, however, identify a clinically consequential subgroup with a pronounced tendency towards peritoneal dissemination.

Systemic anticancer therapy is the standard treatment backbone for metastatic gastric cancer. European and Japanese guidelines recommend fluoropyrimidine–platinum chemotherapy combined, where appropriate, with immune checkpoint inhibition or biomarker-directed therapy; routine gastrectomy, cytoreductive surgery (CRS), hyperthermic intraperitoneal chemotherapy (HIPEC) or other regional strategies is not established for unselected patients with PM (6, 7). Recent specialist consensus statements are more permissive about referral, staging laparoscopy and carefully selected investigational treatment at peritoneal-surface malignancy centres, but they also acknowledge the low certainty and geographic variation in the evidence (8).

The attraction of peritoneal-directed treatment arises from a clinical and immunological problem that is poorly represented by radiological response assessed using the Response Evaluation Criteria in Solid Tumours (RECIST) and whole-patient progression-free survival (PFS). Peritoneal deposits are often thin, plaque-like, miliary or distributed over bowel and mesentery; they may be occult on computed tomography (CT), non-measurable by RECIST and pharmacologically disadvantaged by fibrosis, high interstitial pressure and disorganised vascularity. Ascites, cytological positivity, bowel dysfunction, malnutrition and obstruction may progress without a change in target-lesion diameter. Systemic therapy remains indispensable, but whole-trial response rates cannot be assumed to represent equivalent control of the peritoneal compartment.

The peritoneal environment also warrants direct immune investigation. Studies of patient fluid and tissue samples describe cytotoxic-cell depletion or exhaustion, macrophage enrichment and suppressive soluble mediators (9–13). These findings provide biological context for the clinical observations in ascitic disease, but their ability to predict checkpoint-inhibitor benefit remains unproven (14, 15).

Three locoregional drug-delivery platforms are most developed: CRS with HIPEC, repeated normothermic intraperitoneal and systemic treatment (NIPS), and pressurised intraperitoneal aerosol chemotherapy (PIPAC). None is inherently an immunotherapy. Each can nevertheless alter tumour burden, cell death, antigen exposure, inflammation and treatment access within the cavity, and each can be combined with systemic or direct intraperitoneal immune treatment.

Directed at clinicians and researchers working in gastric cancer, this review treats gastric cancer with established peritoneal metastases (GCPM) primarily as an immune-compartment problem. It examines the biology of immune evasion, the limitations of systemic immunotherapy in peritoneal-predominant and ascitic disease, and the emerging use of PIPAC, NIPS, HIPEC and direct intraperitoneal immune or cellular therapy. The major regional trials are appraised to define the clinical platform on which immune combinations must operate. The central proposition is that future studies must measure the peritoneum directly, incorporate serial immune sampling and isolate the contribution of each regional or immune component.

## Changing epidemiology and the peritoneal tropism of diffuse gastric cancer

### A clinically important Western phenotype

The epidemiological relevance of this subgroup lies in the combination of changing population demographics and its propensity for advanced presentation. Diffuse and poorly cohesive cancers therefore merit specific consideration when planning diagnostic and treatment capacity, even where overall age-standardised gastric cancer incidence is declining (1, 4, 5).

### From diffuse growth to an immune-adapted peritoneal niche

Diffuse or poorly cohesive morphology, signet-ring features, linitis plastica, serosal invasion, nodal burden and positive peritoneal cytology are repeatedly associated with synchronous or metachronous PM (2, 3). Loss of E-cadherin-mediated adhesion, epithelial–mesenchymal plasticity, resistance to anoikis and infiltrative serosal penetration facilitate release into the cavity. Disseminated cells must then survive in ascitic fluid, attach to mesothelium, invade submesothelial matrix and adapt to hypoxia, nutrient stress and immune surveillance (16, 17).

Survival within this environment requires adaptation to local stromal and immune interactions. These processes connect the infiltrative phenotype to the peritoneal immune biology considered below.

## Gastric cancer peritoneal metastases as a distinct immune compartment

### A surface–fluid ecosystem

The peritoneal cavity is not an empty anatomical space. It contains resident macrophages, lymphocyte aggregates, mesothelial cells, adipocytes, fibroblasts, extracellular matrix and soluble mediators. Disseminated gastric-cancer cells must survive detachment and anoikis, circulate as single cells or multicellular aggregates, attach to and retract the mesothelium, invade the submesothelial matrix and acquire a vascular and stromal supply. Inflammatory and wound-healing programmes can convert mesothelium and stroma into a permissive niche; fibroblasts remodel the matrix and raise interstitial pressure, adipocytes provide metabolic substrates, and vascular endothelial growth factor (VEGF)-driven permeability sustains ascites (16, 17).

This architecture creates at least three related but non-identical specimens: solid peritoneal implants, malignant ascites and free intraperitoneal tumour cells. Blood is a fourth, systemic compartment. A biomarker measured in the primary tumour or peripheral blood may therefore fail to represent the cells, ligands and spatial relationships encountered by therapy at the metastatic surface. Conversely, ascites is readily sampled but cannot be assumed to reproduce the immune state inside an established fibrotic nodule. Reliable interpretation requires paired and longitudinal sampling rather than treating any single specimen as a surrogate for the whole disease.

### Loss and exhaustion of cytotoxic lymphocytes

Human studies consistently identify impaired cytotoxic immunity within GCPM. In peritoneal fluid from 122 patients across gastric-cancer stages, patients with PM had lower proportions of CD8-positive T cells, natural killer T-like cells and natural killer cells than patients without PM; CD8-positive T-cell and natural killer T-like-cell proportions fell as the tumour-to-leukocyte ratio increased (9). In paired malignant ascites and blood, ascitic CD4-positive and CD8-positive T cells expressed higher levels of programmed cell death protein 1 (PD-1), T-cell immunoreceptor with Ig and ITIM domains (TIGIT), T-cell immunoglobulin and mucin-domain containing-3 (TIM-3), lymphocyte activation gene 3 (LAG-3) or cytotoxic T-lymphocyte-associated protein 4 (CTLA-4), whilst CD8-positive cells showed an eomesodermin-high/T-bet-low terminally exhausted phenotype (10). These findings imply that the problem is not simply absence of lymphocytes: tumour-reactive cells may be present but functionally constrained by co-inhibitory signalling and terminal differentiation.

Paired primary and peritoneal-tumour tissue provides the spatial counterpart. Peritoneal metastases contained fewer CD4-positive and CD8-positive T cells than matched primary cancers and were commonly classified as immune-desert phenotypes or low-T-cell phenotypes with programmed death-ligand 1 (PD-L1) expression (“intrinsic induction”) (10). In single-cell analysis of malignant ascites, even T-cell-dominant samples contained granzyme-K-positive precursor-exhausted CD8-positive cells rather than a uniformly effective inflamed response (11). These small cohorts do not establish a universal immune state, but they explain why primary-tumour PD-L1 combined positive score (CPS) alone may be an incomplete predictor of peritoneal response.

### Macrophage and myeloid dominance

The second recurrent feature is tumour-promoting myeloid dominance. In peritoneal fluid, CD163-positive M2-like macrophages were markedly enriched in patients with PM, whereas natural killer and cytotoxic T-cell populations were depleted (9). Single-cell sequencing of malignant ascites likewise identified macrophage-rich ecosystems and tumour–macrophage communication through chemokine, matrix and inflammatory pathways (12). “M2” is an imperfect binary label for a continuum of macrophage states, but the convergence across flow cytometry and single-cell studies supports myeloid suppression as a central feature rather than a minor accompaniment.

In 51 patients, analysis of 63 ascites and 43 blood samples identified a cathepsin-high to complement-C1q-high macrophage transition. Recombinant C1q increased PD-L1 and nectin cell adhesion molecule 2 (NECTIN2) expression in ascites-treated gastric cancer cell lines; a recombinant C1 inhibitor reduced expression in macrophage co-cultures (11). These functional experiments support complement-pathway involvement beyond correlation, but pathway inhibition is not C1q-specific genetic knockdown. They do not establish clinical benefit from complement blockade.

### Malignant ascites as an immunosuppressive liquid niche

Malignant ascites is both a clinical phenotype and a liquid tumour microenvironment. Compared with paired plasma or benign cirrhotic ascites, it contains higher concentrations of VEGF, angiopoietin-2, hepatocyte growth factor, matrix metalloproteinases and, for several comparisons, transforming growth factor beta 1 (TGF-β1) and soluble E-cadherin (10). Earlier body-fluid profiling also found higher VEGF-A and interleukin-10, activated or PD-1-positive memory T cells and forkhead box P3-positive regulatory T cells in malignant effusions than in matched blood (13). These mediators can promote vascular leak, matrix remodelling, T-cell dysfunction and tumour-cell survival, whilst the fluid phase permits continual contact between free tumour cells and suppressive immune populations.

Ascites is not biologically uniform. Immune-cell-rich, macrophage-dominant and epithelial-dominant states have been described, and composition can change with treatment. A 2026 single-cell study identified an epithelial–immune dual-phenotype population and derived a 10-gene score associated with anti-PD-1 response; the reported validation discrimination was high, but the discovery set included only ten paired ascites–blood samples and the classifier requires prospective validation (18).

### Stromal, mesothelial and vascular immune exclusion

Immune dysfunction is reinforced by physical and stromal barriers. Dense extracellular matrix, activated fibroblasts, mesothelial remodelling, high interstitial pressure and poorly organised vessels restrict drug distribution and lymphocyte access. VEGF and angiopoietin-2 support ascites and abnormal angiogenesis; Transforming growth factor beta (TGF-β) can couple fibrosis to T-cell exclusion; matrix metalloproteinases facilitate invasion whilst remodelling cytokine and growth-factor availability (10, 17). This provides a rationale for combinations that pair checkpoint blockade with chemotherapy, vascular normalisation or regional drug delivery. The relevant clinical question is whether modifying these barriers improves durable peritoneal and systemic control.

### Spatial heterogeneity and biomarker implications

The clinically relevant immune phenotype may differ between the primary tumour, solid peritoneal deposit, ascitic tumour cell and circulating immune system. PD-L1 can be spatially heterogeneous and dynamic; microsatellite instability (MSI)-high disease is uncommon amongst diffuse peritoneal-predominant cancers; human epidermal growth factor receptor 2 (HER2) expression may be lost across metastatic sites; and claudin 18 isoform 2 (CLDN18.2) expression can coexist with diffuse histology and peritoneal dissemination. Trials should therefore record where and when each biomarker was measured and, when feasible, compare archival primary tissue with contemporaneous peritoneal tissue or ascites cell blocks.

**Table 1** summarises the principal human observations, their therapeutic implications and their limitations. The evidence is compelling enough to justify compartment-specific investigation, but not to label every patient with GCPM as immunotherapy-resistant. Most studies are small, retrospective, geographically concentrated and enriched for patients with clinically accessible ascites. Prior treatment, disease burden and sampling technique are important confounders.

**Table 1:** The immune microenvironment of gastric cancer peritoneal metastases: human evidence and implications.

Domain	Direct human observations	Mechanism of immune evasion	Therapeutic or biomarker implication	Limitations
Cytotoxic T cells	Ascitic T cells express PD-1, TIGIT, TIM-3, LAG-3 and/or CTLA-4; CD8 cells show terminal or precursor exhaustion. Peritoneal deposits have lower CD4/CD8 density than paired primary tumours (10, 11).	Multiple co-inhibitory axes and terminal differentiation may limit reinvigoration by PD-1 blockade alone.	Measure T-cell state, not only abundance; test rational multi-axis or myeloid-directed combinations cautiously.	Small retrospective cohorts; prior therapy and ascites accessibility introduce selection.
NK and NKT-like cells	Peritoneal fluid from patients with PM contains fewer NK and NKT-like cells than fluid from patients without PM (9).	Reduced innate cytotoxic surveillance may facilitate survival of free tumour cells.	Include NK/NKT phenotyping and functional assays; supports trials such as IL-2-based local immune stimulation.	Cross-sectional phenotype; functionality and causal relevance remain incompletely defined.
Macrophage/myeloid compartment	CD163-positive M2-like macrophages are enriched; single-cell studies show macrophage-dominant ascites and a CTS-high to C1Q-high TAM transition (9, 11, 12).	Experiments support complement-pathway involvement in PD-L1 and NECTIN2 upregulation on ascitic tumour cells; macrophage-derived chemokine/matrix signals support dissemination.	Evaluate complement, macrophage and TIGIT-axis biomarkers alongside PD-1; paired on-treatment ascites is attractive.	Macrophage states are continuous rather than binary; no validated macrophage-directed therapy in GCPM.
Regulatory and memory T cells	Malignant effusions are enriched for FOXP3-positive Tregs and activated/PD-1-positive memory T cells versus blood (13).	Regulatory suppression can coexist with apparent immune activation, making “immune-rich” ascites potentially dysfunctional.	Functional and clonotypic assessment may be more informative than simple cell counts.	Mixed body-fluid sites and modest sample size.
Soluble ascitic factors	VEGF, angiopoietin-2, HGF, MMPs, IL-10 and/or TGF-β-related signals are elevated in malignant ascites in paired or benign-fluid comparisons (10, 13).	Vascular leak, matrix remodelling, fibrosis and suppression create a protected liquid niche.	Rationale for vascular-normalising or stroma-modifying combinations; measure ascites kinetics and soluble mediators.	Concentration does not prove pathway dependence; fluid dilution and processing affect results.
Stroma, mesothelium and vasculature	Paired tissue shows immune-desert/intrinsic-induction phenotypes; peritoneal implants are often fibrotic and poorly vascularised (10, 17).	Physical exclusion and high interstitial pressure restrict drug and immune-cell penetration.	Spatial biopsy and distribution-aware PCI should accompany systemic and regional studies.	Direct human spatial datasets remain small; much mechanistic evidence is preclinical.
Ascitic tumour-cell adaptation	Ascites-disseminated cells can express PD-L1 and NECTIN2 and display epithelial–mesenchymal or dual epithelial–immune states (11, 18).	Checkpoint-ligand expression, plasticity and spheroid survival permit immune escape in suspension.	Ascites cell blocks and single-cell profiling may complement primary-tumour biomarkers.	Candidate signatures require prospective, external validation.
Spatial and temporal heterogeneity	Primary tumour, peritoneal deposit, ascites and blood show discordant immune-cell density and state; treatment changes composition (10, 13).	A single archival biopsy can misclassify the active metastatic niche.	Use paired baseline/on-treatment blood, fluid and tissue; report site and timing of biomarker testing.	Repeated laparoscopy is invasive and serial samples are vulnerable to survivorship bias.

C1Q, complement component 1q; CTS, cathepsin; GCPM, gastric cancer peritoneal metastases; HGF, hepatocyte growth factor; MMP, matrix metalloproteinase; NECTIN2, nectin cell adhesion molecule 2; NK, natural killer; NKT, natural killer T; PD-1, programmed cell death protein 1; PD-L1, programmed death-ligand 1; PM, peritoneal metastases; TAM, tumour-associated macrophage; TGF-β, transforming growth factor beta; TIGIT, T-cell immunoreceptor with Ig and ITIM domains; Treg, regulatory T cell; VEGF, vascular endothelial growth factor.

### Pharmacology and the rationale for regional exposure

The pharmacological argument for regional treatment is similarly specific. Intraperitoneal administration can produce a high cavity-to-plasma exposure ratio for drugs whose molecular properties favour retention, particularly taxanes. Paclitaxel remains in the peritoneal cavity for prolonged periods and penetrates superficial tumour nodules, whilst concomitant systemic therapy treats vascularised tumour and occult extra-peritoneal disease (16). However, high cavity concentration does not ensure homogeneous penetration through bulky, fibrotic or poorly exposed implants. This is why NIPS is conceptually most persuasive as repeated treatment of small-volume or treatment-responsive deposits, and why HIPEC is biologically intended for microscopic residual disease after complete macroscopic cytoreduction rather than as compensation for incomplete surgery.

Regional chemotherapy may change antigen exposure and local inflammation, whilst checkpoint inhibition may restore activity in responsive lymphocyte populations. The tissue findings in the phase I PIANO study of PIPAC oxaliplatin and systemic nivolumab support investigation of this interaction (19), but neither pharmacodynamic change nor activity in a single combination arm demonstrates synergy. Here, synergy denotes an interaction exceeding the expected effects of the individual treatments under a prespecified model. Its assessment requires appropriate component comparators and an interaction analysis; demonstrating benefit from adding one treatment establishes incremental efficacy, not necessarily synergy.

### Why conventional response assessment is insufficient

CT has limited sensitivity for small-volume PM, particularly along the small-bowel serosa, mesentery and subphrenic spaces. A systematic review found substantial variation in the performance of CT, positron emission tomography/CT and magnetic resonance imaging and confirmed the continuing value of staging laparoscopy for detecting occult disease (20). Radiological non-progression is therefore a weak biological filter before a high-risk regional intervention. A patient may have no new measurable lesion but still have an increasing Peritoneal Cancer Index (PCI), persistent positive cytology or progressive mesenteric disease that makes complete cytoreduction impossible.

The PCI improves anatomical description by combining lesion size across 13 abdominopelvic regions, but a single total score remains incomplete. PCI 6 confined to accessible parietal surfaces differs technically from PCI 6 involving the small-bowel mesentery. Cytology captures free tumour cells but is influenced by lavage technique, sampling and processing. Ascites volume reflects both tumour biology and host physiology. Histological response scores can demonstrate regression after NIPS or PIPAC, although different systems and biopsy practices limit cross-trial comparison (21). A robust peritoneal response assessment should therefore combine PCI trajectory, anatomical distribution, cytology, ascites, representative biopsy and technical assessment of potential complete macroscopic cytoreduction (CC-0), with no visible residual peritoneal disease.

This multidimensional approach also clarifies the meaning of “conversion.” Conversion should not mean that surgery became technically possible after any period without overt progression. For PM, it should denote a sustained transition to a biologically and anatomically favourable state: no extra-peritoneal progression; clearance or substantial control of ascites; cytological conversion where initially positive; major reduction in PCI; preserved nutrition and performance status; and a high probability of both CC-0 peritoneal cytoreduction and microscopically margin-negative gastric resection (R0).

## Systemic and combination immunotherapy: progress and the peritoneal evidence gap

### Checkpoint inhibition plus chemotherapy

Modern first-line therapy is determined by HER2 status, PD-L1 expression, mismatch-repair (MMR)/MSI status, CLDN18.2 expression and patient fitness. Fluoropyrimidine–platinum chemotherapy remains the common platform, with immune checkpoint inhibition for appropriate HER2-negative disease, trastuzumab-based treatment for HER2-positive disease and zolbetuximab for CLDN18.2-positive/HER2-negative disease where available (6, 7). These advances are clinically important and must be incorporated into any future peritoneal-directed trial. The central problem is not absence of systemic efficacy, but absence of sufficiently granular peritoneal reporting.

The randomised phase III CheckMate 649 trial compared nivolumab plus chemotherapy with chemotherapy alone and established nivolumab plus chemotherapy as a first-line standard in HER2-negative advanced gastric, gastro-oesophageal junction and oesophageal adenocarcinoma. In patients with PD-L1 CPS ≥5, median overall survival (OS) was 14.4 versus 11.1 months (hazard ratio [HR] 0.71), and five-year follow-up confirmed a durable survival tail (22, 23). A subgroup recorded as having peritoneal metastases had a median OS of 14.8 months with nivolumab plus chemotherapy. However, this was not a peritoneal-only cohort; concurrent metastatic sites, PCI, cytology, ascites and laparoscopic response were not characterised. The subgroup supports use of nivolumab according to established indications, not an inference that immunochemotherapy reliably converts PM to an operable state.

The randomised phase III KEYNOTE-859 trial similarly showed that pembrolizumab plus chemotherapy improved median OS from 11.5 to 12.9 months in the intention-to-treat population (HR 0.78), with a larger effect at CPS ≥10 (15.7 versus 11.8 months; HR 0.65) (24). It did not report a dedicated peritoneal efficacy analysis. For HER2-positive disease, the final analysis of the randomised phase III KEYNOTE-811 trial reported median OS of 20.0 versus 16.8 months with pembrolizumab added to trastuzumab and chemotherapy (HR 0.80), with benefit concentrated principally in PD-L1-positive tumours (25). Again, PCI, cytology, ascites response and peritoneal-specific progression were not reported.

These pivotal trials establish standards of care but do not resolve whether immunochemotherapy has the same relative effect in peritoneal-predominant disease, particularly when malignant ascites is present. In a retrospective single-centre cohort, 339 patients received first-line nivolumab plus chemotherapy and 422 received chemotherapy. Within the nivolumab cohort, patients with PM and malignant ascites had median PFS and OS of 5.1 and 11.6 months, compared with 10.8 and 19.7 months in PM without ascites and 10.2 and 22.0 months without PM. Addition of nivolumab was not associated with a statistically significant survival advantage over chemotherapy in the ascitic subgroup (14). Because treatment was not randomised and ascites marks high burden and poor physiology, this study cannot distinguish predictive resistance from adverse prognosis. It does identify malignant ascites as a necessary stratification variable.

A multicohort study in mismatch-repair-deficient/microsatellite-instability-high colorectal and gastric cancers reached a compatible conclusion: PM with ascites was associated with shorter survival on immune checkpoint inhibition, including in a 59-patient gastric-cancer validation cohort (15). This is notable because MSI-high status is otherwise strongly predictive of checkpoint benefit, but residual prognostic confounding remains. Smaller clinical series show that nivolumab plus modified FOLFOX6 (mFOLFOX6; fluorouracil, leucovorin and oxaliplatin) can improve ascites or oral intake in some patients with severe PM (26). These observations support treatment according to established indications whilst assessing peritoneal response directly (14, 15, 26).

### Biomarker-directed combinations and cellular therapy

CLDN18.2 is especially relevant because positive cancers often display diffuse histology and peritoneal dissemination. In the randomised phase III SPOTLIGHT trial, zolbetuximab plus mFOLFOX6 improved median PFS from 8.67 to 10.61 months and median OS from 15.54 to 18.23 months (both HR 0.75) (27). In the randomised phase III GLOW trial, zolbetuximab plus capecitabine and oxaliplatin (CAPOX) improved median PFS from 6.80 to 8.21 months (HR 0.69) and median OS from 12.16 to 14.39 months (HR 0.77) (28). Neither trial reported outcomes specifically amongst patients with PM. Moreover, the OS improvement in GLOW was not accompanied by a large objective-response-rate increment (42.5% versus 40.3%), emphasising that survival benefit across a metastatic population is not equivalent to deep anatomical conversion.

Cohort 4B of the phase II ILUSTRO study, a single-arm investigation, combined zolbetuximab, nivolumab and mFOLFOX6 in 71 patients with untreated CLDN18.2-positive, HER2-negative advanced disease. Median PFS was 14.8 months and the objective response rate was 62.1% amongst 58 patients with measurable disease; OS was immature (29). These findings suggest that more active systemic combinations may increase the number of profound responders. They still do not establish how frequently such treatment clears cytology, reduces PCI or controls diffuse non-measurable implants.

CLDN18.2 has also become an immune-cell target. In the randomised phase II CT041-ST-01 trial, the chimeric antigen receptor (CAR) T-cell therapy satricabtagene autoleucel (satri-cel) improved median PFS from 1.77 to 3.25 months versus physician-choice therapy in heavily pretreated CLDN18.2-positive gastric or gastro-oesophageal-junction cancer (HR 0.37). PM was present in 69% and 60% of the groups, respectively, but peritoneal-specific efficacy was not reported; grade 3 or worse treatment-emergent adverse events were near universal and cytokine-release syndrome occurred in most treated patients (30). Satri-cel therefore validates CLDN18.2-directed cellular therapy in systemic disease, not a PM-specific effect. It also provides a strong rationale for studying trafficking and persistence in ascites and peritoneal tissue rather than blood alone.

### PM-specific conversion and anti-angiogenic combinations

Dedicated PM cohorts are beginning to test combination immunotherapy more directly. In a single-arm phase II trial of toripalimab (an anti-PD-1 antibody) plus fluorouracil, leucovorin, oxaliplatin and docetaxel (FLOT) in 20 patients with laparoscopically proven PM, objective response and disease-control rates were 35% and 80%; five patients underwent R0 conversion surgery. Median PFS and OS were 6.5 and 10.8 months and grade 3–4 treatment-related adverse events occurred in 35% (31). The small operated subset and absence of a comparator prevent attribution of benefit to toripalimab or surgery, but the trial illustrates the value of requiring laparoscopic confirmation rather than relying on a mixed metastatic subgroup.

Checkpoint blockade combined with anti-angiogenic therapy is attractive because VEGF contributes to ascites, abnormal vasculature and immune suppression. A 30-patient multicentre retrospective study of anti-PD-1 therapy, a small-molecule anti-angiogenic drug and chemotherapy reported median time to treatment failure of 7.03 months and OS of 13.33 months; amongst 12 patients with measurable disease, the response rate was 58.3%. Patients with ascites had shorter time to failure and OS than those without ascites (32). Regimens and staging methods were heterogeneous and only a minority had laparoscopically confirmed PM. This is a signal-generating strategy, not evidence that vascular–immune synergy has overcome the ascitic phenotype.

### What systemic trials should report

The reporting gap is systematic (**Table 2**). Pivotal trials commonly record PM as a baseline metastatic site but rarely distinguish isolated PM from multi-organ disease, macroscopic disease from cytology-only disease, or ascitic from non-ascitic presentations. RECIST response usually reflects measurable lesions that may lie outside the peritoneum. Consequently, the evidence required to select patients for a peritoneal intervention is missing even when the systemic regimen clearly improves OS.

**Table 2:** Systemic and regional immunotherapy evidence relevant to gastric cancer peritoneal metastases.

Study/design	Regimen and route	PM/ascites definition	Principal findings	Immune or peritoneal information	Interpretation
CheckMate 649; phase III RCT (22, 23)	IV nivolumab + chemotherapy vs chemotherapy	Baseline PM recorded; not PM-only	CPS ≥5 OS 14.4 vs 11.1 months; durable 5-year tail	Nivolumab-combination median OS 14.8 months in recorded PM subgroup; no PCI/cytology/ascites data	Supports standard systemic ICI; does not establish peritoneal conversion or ascites-specific benefit.
KEYNOTE-859/KEYNOTE-811; phase III RCTs (24, 25)	IV pembrolizumab + chemotherapy, with trastuzumab in HER2-positive disease	PM not analysed as a dedicated compartment	OS benefit in overall biomarker-defined populations	No PCI, cytology, ascites response or peritoneal-first progression	Registration efficacy cannot be translated directly into compartment-specific control.
Lee et al.; retrospective comparative cohort (14)	First-line nivolumab + chemotherapy; separate chemotherapy cohort	PM stratified by malignant ascites	Nivolumab cohort PM+ascites: PFS 5.1 and OS 11.6 months; no significant added benefit vs chemotherapy in this subgroup	Ascites identifies an adverse phenotype despite ICI	Important stratification signal; non-random treatment assignment and prognostic confounding.
Fucà et al.; multicohort retrospective (15)	Immune checkpoint inhibition	dMMR/MSI-H CRC and GC; PM with/without ascites	PM with ascites associated with shorter PFS/OS; finding reproduced in 59-patient GC cohort	Suggests ascitic suppression may persist in an ICI-sensitive molecular group	Cannot completely separate predictive resistance from adverse prognosis.
Toripalimab–FLOT; single-arm phase II, n=20 (31)	IV toripalimab + FLOT; conversion surgery considered	Laparoscopically proven GCPM	ORR 35%, DCR 80%, PFS 6.5, OS 10.8 months; R0 surgery 5/20	No serial immune profiling reported	PM-specific feasibility; no comparator and very small selected surgical subset.
PIANO; phase I, n=18 (19)	PIPAC oxaliplatin + systemic nivolumab	Previously treated GCPM	Feasible; repeat-procedure subsets showed PCI/PRGS improvement	43 samples: inflammatory/immune signatures increased, M2-like macrophages decreased, memory T-cell populations increased	Strong pharmacodynamic signal; cannot isolate PIPAC, nivolumab or synergy.
IPLUS; single-arm phase II, n=22 (41)	IP paclitaxel + FOLFOX ± systemic nivolumab	Histologically confirmed PM; no haematogenous metastases	PFS 13.1, OS 20.2 months; conversion surgery 9/22	Nivolumab optional (13/22) and not independently associated with outcome	Feasibility signal; no randomised ICI or surgery comparison.
HIPEC–camrelizumab conversion; single-arm phase II, n=46 (42)	Laparoscopic paclitaxel HIPEC then systemic camrelizumab + paclitaxel/S-1	PM, PCI ≤20, no other distant disease	Surgery 31/46; overall R0 28/46; grade ≥3 treatment-related toxicity 19.6%	Peritoneal response reported; no component-specific immune analysis	Abstract-level multimodal signal; surgery comparison is response-selected.
Intraperitoneal nivolumab; pilot, n=9 (34)	IP nivolumab 20 mg after paracentesis	Mixed GI/pancreaticobiliary malignant ascites; gastric cancer n=2	Clinical ascites response 7/9; limited local toxicity	Ascitic tumour-cell reduction correlated with response	Proof-of-concept for local delivery only; no systemic efficacy inference.
Satri-cel CT041-ST-01; phase II RCT, n=156 (30)	IV CLDN18.2 CAR T cells vs physician-choice therapy	PM in 69% vs 60%; not PM-specific	PFS 3.25 vs 1.77 months, HR 0.37	Peritoneal-specific response/trafficking not reported in the RCT	Validates systemic cellular therapy; local immune relevance remains unresolved.

CAR, chimeric antigen receptor; CLDN18.2, claudin 18 isoform 2; CPS, combined positive score; CRC, colorectal cancer; GC, gastric cancer; DCR, disease-control rate; dMMR, mismatch-repair deficient; FLOT, fluorouracil, leucovorin, oxaliplatin and docetaxel; GCPM, gastric cancer peritoneal metastases; GI, gastrointestinal; HIPEC, hyperthermic intraperitoneal chemotherapy; HR, hazard ratio; ICI, immune checkpoint inhibitor; IP, intraperitoneal; IV, intravenous; MSI-H, microsatellite-instability high; ORR, objective response rate; OS, overall survival; PCI, Peritoneal Cancer Index; PFS, progression-free survival; PIPAC, pressurised intraperitoneal aerosol chemotherapy; PM, peritoneal metastases; PRGS, peritoneal regression grading score; RCT, randomised controlled trial.

A prospectively identified PM cohort would allow treatment effects to be related to disease distribution and ascites burden. The common clinical dataset and paired immune-sampling strategy proposed below provide a framework for this assessment.

## Peritoneal-directed treatment as an immunological platform

### Direct intraperitoneal immunotherapy and cellular therapy

The most conceptually direct strategy is to place an immune agent inside the cavity. This may increase local exposure, bypass inefficient trafficking into peritoneal deposits and permit pharmacodynamic sampling from ascites. It cannot, however, replace systemic control of occult haematogenous disease, and local delivery still encounters antigen heterogeneity, T-cell exhaustion, suppressive myeloid cells and fibrotic barriers.

Catumaxomab, a trifunctional antibody targeting epithelial cell adhesion molecule (EpCAM) and CD3, was administered intraperitoneally before FLOT in a small randomised phase II gastric-cancer study. Amongst 31 evaluable patients, macroscopic complete response was 27% versus 19%, median PFS 6.7 versus 5.4 months and median OS 13.2 versus 13.0 months; the trial stopped early after the drug became unavailable and toxicity included severe nausea, infection, pain and liver-enzyme elevation (33). The study remains important as proof that local T-cell engagement can be delivered multicentrically, but it did not establish efficacy.

A 2026 pilot administered intraperitoneal nivolumab after paracentesis to nine patients with malignant ascites from mixed gastrointestinal and pancreaticobiliary cancers, only two of whom had gastric cancer. Seven achieved a prespecified clinical ascites response with limited local toxicity (34). The cohort is too small and heterogeneous to support systemic efficacy claims. The phase Ib CONTROL trial combines intraperitoneal aldesleukin on days 1 and 8 with systemic nivolumab and FOLFOX (fluorouracil, leucovorin and oxaliplatin) in gastric or gastro-oesophageal junction cancer confined to the peritoneum and incorporates serial blood, peritoneal fluid and tissue assessment of T-cell, natural-killer-cell and regulatory populations (35).

Regional cellular therapy is earlier still. Preclinical gastro-oesophageal models suggested that intraperitoneal mesothelin-directed CAR T cells achieved superior peritoneal control to intravenous delivery at lower dose; a first-in-human phase I study (NCT06623396) is evaluating intraperitoneal mesothelin CAR T cells in mesothelin-positive gastro-oesophageal adenocarcinoma with peritoneal carcinomatosis (36). This programme directly tests whether regional delivery can improve cellular trafficking, but must also address persistence within suppressive ascites and control beyond the cavity.

### PIPAC as a platform for serial immune interrogation

PIPAC delivers aerosolised chemotherapy during laparoscopy under pressure and can be repeated. Its immunological attraction is not simply regional drug concentration: each procedure permits direct inspection, PCI documentation and protocolised biopsy, allowing immune change to be linked to histological and anatomical response. Most gastric-cancer evidence remains phase I/II or registry-based, with substantial attrition because progression, obstruction or adhesions prevent repeat procedures (37–39).

The phase I PIANO trial investigated PIPAC oxaliplatin with systemic nivolumab and included serial peritoneal tissue profiling. Eighteen previously treated patients received PIPAC oxaliplatin 90 mg/m² every six weeks and nivolumab 240 mg every two weeks. In the smaller repeat-procedure subsets, median PCI decreased by five points at the second and seven at the third PIPAC, whilst a peritoneal regression grading score (PRGS) of 1–2 occurred in six of nine and all three evaluable patients. Analysis of 43 peritoneal samples showed enrichment of inflammatory, immune and stromal signatures, reduction in M2-like macrophages and increases in memory CD4-positive and CD8-positive populations (19). These observations demonstrate local immune remodelling, but the absence of PIPAC-only and nivolumab-only groups prevents any claim of clinical synergy.

The phase III PIPAC VER-One trial randomises low-volume PM or positive cytology to FOLFOX with or without repeated cisplatin–doxorubicin PIPAC (40). It is not an immunotherapy trial, but its repeated-access design could establish the biological and clinical platform on which a later immune combination should be tested.

### NIPS and repeated intraperitoneal paclitaxel with systemic immunotherapy

NIPS repeatedly delivers intraperitoneal chemotherapy, most commonly paclitaxel, through an implanted port whilst systemic therapy continues. The pharmacological advantage is sustained cavity exposure to a taxane with favourable peritoneal retention; a potential practical advantage is maintaining systemic-treatment continuity without the recovery period required by major surgery. Serial laparoscopy and cytology can convert therapy into a longitudinal biological test rather than a single locoregional event (16).

The phase II IPLUS study combined intraperitoneal paclitaxel 60 mg/m² with FOLFOX, with nivolumab received by 13 of 22 patients. Median PFS and OS were 13.1 and 20.2 months; nine patients underwent conversion surgery and their median OS was 32.9 months. Nivolumab exposure was optional and not significantly associated with PFS or OS, whilst surgery was response-selected (41). IPLUS supports feasibility of integrating a Western systemic backbone, checkpoint inhibition and intraperitoneal paclitaxel, but cannot identify the incremental immune contribution.

### CRS and HIPEC as immune perturbations

CRS and HIPEC create a different biological intervention: abrupt macroscopic tumour removal followed by heated regional chemotherapy intended for free cells and superficial microscopic residual disease. Debulking, heat and chemotherapy may release antigen and alter the suppressive tumour reservoir, but major surgery can also cause lymphopenia, inflammation, nutritional loss and delayed systemic treatment. These competing immune and clinical effects make postoperative recovery and systemic-treatment resumption part of the mechanistic question, not merely perioperative quality indicators.

A prospective phase II study combined two paclitaxel HIPEC procedures with systemic camrelizumab, paclitaxel and S-1 (the oral combination of tegafur, gimeracil and oteracil) in 46 patients with PM and no other distant disease; 31 proceeded to surgery and 28 achieved R0 resection. The comparison between operated and non-operated patients is dominated by response selection, and the effects of HIPEC, checkpoint inhibition and surgery cannot be separated (42). HISTORIA similarly studies paclitaxel HIPEC followed by tislelizumab and S-1 plus oxaliplatin (SOX) in a prospective single-arm pathway (43). These studies test feasible multimodal packages; they do not yet prove that HIPEC enhances checkpoint efficacy.

## What established regional trials contribute to the immune-therapy question

### Repeated intraperitoneal paclitaxel establishes an active regional platform

The randomised phase III PHOENIX-GC trial evaluated repeated intraperitoneal paclitaxel in gastric cancer with PM. Intraperitoneal plus intravenous paclitaxel with S-1 did not significantly improve the primary OS analysis over S-1/cisplatin (17.7 versus 15.2 months; HR 0.72; P = 0.080), although an ascites-adjusted analysis and three-year survival favoured the intraperitoneal strategy (44). Different systemic backbones limited causal isolation, but the trial established a credible regional signal.

The randomised phase III DRAGON-01 trial more directly isolated the route of paclitaxel delivery by comparing S-1 plus intravenous paclitaxel with the same oral therapy and a matched total paclitaxel dose divided between intravenous and intraperitoneal delivery. In 222 treated patients with laparoscopically confirmed PM and no extra-peritoneal distant disease, median OS improved from 13.9 to 19.4 months (HR 0.67), PFS from 7.2 to 11.2 months (HR 0.72), and cytological conversion from 52.6% to 83.6% (45). This is the strongest evidence that deliberate treatment of the cavity can change outcome. PD-L1 and MSI/MMR status were unavailable, so the value of adding intraperitoneal paclitaxel to contemporary immunochemotherapy remains an open question.

The single-arm phase II STOPGAP trial evaluated sequential systemic treatment and intraperitoneal paclitaxel in a Western population. After three to six months without progression on systemic therapy, 31 patients received four cycles of intraperitoneal and intravenous paclitaxel with fluorouracil/leucovorin; previously indicated anti-PD-1 therapy or trastuzumab could continue. Thirty completed treatment, 11 underwent CC-0 CRS–HIPEC, and median OS from diagnosis was 17.5 months overall and 38.0 months amongst operated patients (46, 47). The operated comparison is profoundly response-selected, but the pathway demonstrates how sustained systemic control, regional therapy and repeat laparoscopy can identify a clinically responding population before surgery.

### Operative trials define constraints rather than immune efficacy

The principal operative trials answer a different question. The randomised phase III GASTRIPEC-I trial found no OS improvement from adding mitomycin C–cisplatin HIPEC to an intended CRS pathway, although PFS improved; only 50 of 105 randomised patients underwent protocol CRS and only 36 restarted postoperative chemotherapy (48). The randomised phase III RENAISSANCE trial found no benefit from early radical surgery across heterogeneous limited-metastatic disease after four FLOT cycles, with 8% 90-day mortality and reduced post-randomisation chemotherapy exposure; the small peritoneal subgroup had median OS of 12 versus 19 months with surgery and continued systemic therapy, respectively (49, 50). Neither trial evaluated an immune-selected population or a regional immune strategy.

The randomised phase III PERISCOPE II trial is most relevant as a caution about multicomponent treatment and opportunity cost. After at least three systemic-treatment cycles without progression, it compared continued therapy with gastrectomy, CRS, heated oxaliplatin and normothermic docetaxel in low-volume PM or positive cytology. Median OS was 16.6 versus 15.7 months (HR 1.10); only 37 of 50 patients assigned to the operative pathway completed protocol treatment, serious morbidity was substantial, three treatment-related deaths occurred and only eight had restarted their baseline regimen within 13 weeks (51, 52).

The results do not support routine use of this early operative package. For this review, its broader lesson is that a locoregional intervention cannot be judged by technical clearance alone: it must preserve immune and systemic treatment opportunity. PERISCOPE II did not test sustained intraperitoneal paclitaxel, direct intraperitoneal immunotherapy, or surgery after deep systemic, cytological and immune response. Those questions require different designs, not reanalysis of its operated subgroup. **Table 3** places the completed trials in their appropriate causal categories.

**Table 3:** Completed studies defining the clinical evidence for peritoneal-directed treatment.

Study/design	Population and selection	Intervention/comparator	Principal efficacy findings	Peritoneal, surgical and delivery findings	Interpretation
PHOENIX-GC; phase III RCT (44)	Gastric PM; 183 enrolled, 164 eligible for efficacy.	IP + IV paclitaxel + S-1 vs S-1/cisplatin.	OS 17.7 vs 15.2 months, HR 0.72, P = 0.080; 3-year OS 21.9% vs 6.0%. Ascites-adjusted sensitivity HR 0.59.	Baseline ascites imbalance; different systemic backbones.	Primary endpoint negative but generated a credible NIPS efficacy signal.
DRAGON-01; phase III RCT, n=238 randomised/222 treated (45)	Laparoscopically confirmed PM, no extra-peritoneal distant disease; median PCI 15 vs 14.	IP paclitaxel 20 + IV paclitaxel 50 mg/m² + S-1 vs IV paclitaxel 70 mg/m² + S-1.	OS 19.4 vs 13.9 months, HR 0.67; PFS 11.2 vs 7.2 months, HR 0.72; 3-year OS 25.0% vs 12.2%.	Cytology conversion 83.6% vs 52.6%; conversion surgery 50.7% vs 35.1%; operated OS 33.1 vs 18.8 months; port complications 27.0%.	Positive randomised evidence for sustained IP paclitaxel. Surgery was not randomised and did not include HIPEC.
STOPGAP; single-arm phase II, n=31 (46, 47)	Gastric/Siewert III PM without visceral metastases; no progression after 3–6 months systemic therapy; median PCI 8.	Four cycles IP paclitaxel 40 + IV paclitaxel 50 mg/m² + 5-FU/leucovorin; repeat laparoscopy; selected CRS–HIPEC.	12-month PFS 28%; OS 17.5 months from diagnosis and 11.1 months from study entry.	30 completed NIPS; 11 underwent CC-0 CRS–HIPEC; operated vs non-operated OS 38.0 vs 13.9 months from diagnosis.	Western feasibility of longitudinal response selection; single-arm operated comparison cannot establish surgical benefit.
GASTRIPEC-I; phase III RCT, n=105 (48)	Synchronous PM; PCI ≤6 in 46.7%, 7–13 in 36.2%, >13 in 17.1%; randomised before chemotherapy.	Intended CRS vs intended CRS + mitomycin C/cisplatin HIPEC.	OS 14.9 months in both; PFS 3.5 vs 7.1 months; distant-metastasis-free survival 9.2 vs 10.2 months.	Only 50 underwent protocol CRS; CC-0 in 35; 36 restarted postoperative chemotherapy after median 53 days.	No OS benefit from adding HIPEC across the intended CRS population; does not compare CRS pathway with systemic therapy.
RENAISSANCE/AIO-FLOT5; phase III RCT, n=139 (49, 50)	Heterogeneous limited-metastatic disease; four FLOT cycles; no mandatory repeat laparoscopy.	Continued systemic therapy vs radical surgery/local treatment of all sites then postoperative therapy.	25% OS quantile: 14 versus 10 months favouring control numerically; PM subgroup 19 vs 12 months.	Surgery 91%; 90-day mortality 8%; ≥4 post-randomisation cycles 71% vs 42%. PM PCI/CC insufficiently reported; no standard HIPEC.	Warns against early pan-oligometastatic surgery; not a direct CRS–HIPEC trial in isolated responding PM.
PERISCOPE II; phase III RCT, n=101 ITT (51, 52)	cT3–4a, CY+ and/or PCI <7, no extra-peritoneal disease; randomised after ≥3 cycles without progression.	Continued systemic therapy vs gastrectomy + CRS + heated oxaliplatin 460 mg/m² then normothermic docetaxel 50 mg/m².	OS 16.6 vs 15.7 months, HR 1.10; PFS 7.6 vs 9.3 months, HR 0.93.	Only 37/50 completed protocol operation; complications 32/37, leak 10/37, R1 9/37; three treatment deaths; 8/50 restarted baseline therapy within 13 weeks.	Does not support routine use of this early multicomponent strategy; does not test sustained NIPS or surgery after exceptional response.

CC, completeness of cytoreduction; CRS, cytoreductive surgery; CY+, tumour-positive peritoneal cytology; FLOT, fluorouracil, leucovorin, oxaliplatin and docetaxel; HIPEC, hyperthermic intraperitoneal chemotherapy; HR, hazard ratio; IP, intraperitoneal; ITT, intention to treat; NIPS, normothermic intraperitoneal and systemic treatment; OS, overall survival; PCI, Peritoneal Cancer Index; PFS, progression-free survival; PM, peritoneal metastases; RCT, randomised controlled trial.

## Future trials: from immune rationale to causal testing

### Establishing the regional-treatment platform

The first unresolved question is whether deliberate treatment of the cavity adds benefit to optimal biomarker-directed systemic therapy. EA2234/STOPGAP II addresses this in North America after three to six months of first-line treatment without major progression. Patients undergo laparoscopy, cytology and PCI assessment before randomisation to continued physician-choice systemic therapy or intravenous plus intraperitoneal paclitaxel with fluorouracil/leucovorin; indicated immune or targeted therapy may continue (53, 54). This design isolates the intraperitoneal component more clearly than an early operative package.

IPa-Gastric evaluates intraperitoneal paclitaxel with contemporary systemic treatment in a Western phase III setting, whilst PIPAC VER-One evaluates a repeated PIPAC-containing pathway in low-volume disease (40, 55). These studies extend randomised evaluation across different regional-delivery strategies.

Cytoreduction may occur in either STOPGAP II group, and the public protocol does not mandate or standardise HIPEC. The trial can therefore determine the value of adding intraperitoneal paclitaxel within a response-selected pathway, but cannot independently determine the contribution of surgery. This distinction is essential: survival amongst patients who ultimately undergo surgery is a post-randomisation biological-selection signal, not a randomised surgical comparison.

### Trials that directly manipulate peritoneal immunity

The ongoing immune-focused programmes are summarised in **Table 4**. CONTROL, HISTORIA and the intraperitoneal mesothelin CAR T-cell programme examine local cytokine treatment, regional chemotherapy with checkpoint inhibition and regional cellular therapy, respectively (35, 36, 43). Their distinct mechanisms require intervention-specific pharmacodynamic assessments.

**Table 4:** Ongoing trials and the causal questions they can answer.

Trial	Eligibility and timing	Intervention/comparison	Key endpoints and translational design	What the trial will and will not establish
EA2234/STOPGAP II; phase II/III; NCT07001748 (53, 54)	MSS/pMMR gastric or Siewert III PM/CY+; no major progression after 3–6 months first-line therapy; laparoscopy, cytology and PCI before randomisation.	Physician-choice systemic therapy vs IV paclitaxel + IP paclitaxel + 5-FU/leucovorin; indicated immune/targeted therapy may continue.	Phase II PFS; phase III OS; toxicity and peritoneal outcomes.	Tests addition of IP paclitaxel after systemic selection. Cytoreduction may occur in either arm; public materials do not mandate HIPEC.
PIPAC VER-One; phase III; NCT05303714 (40)	Synchronous CY+ and/or PCI ≤6; no extra-peritoneal metastases.	FOLFOX vs FOLFOX + three cisplatin/doxorubicin PIPAC procedures, with multimodality surgical pathway.	Secondary resectability; OS, PFS, histological response, QoL, complications and cost-effectiveness.	Tests a PIPAC-containing pathway; differing surgical policy may complicate isolation of PIPAC.
IPa-Gastric; phase III; NCT07304271 (55)	Western gastric/GEJ PM; laparoscopy and PCI; no other distant disease except ovaries.	Contemporary systemic therapy ± IP paclitaxel.	OS; PFS, EORTC QoL, ascites, conversion surgery and toxicity.	Direct Western test of IP paclitaxel; surgery remains a downstream non-randomised outcome.
CONTROL; phase Ib; NCT05802056 (35)	Gastric/GEJ adenocarcinoma with PM only; PCI 1–24.	IP aldesleukin on days 1/8 + systemic nivolumab/FOLFOX.	PCI reduction; PRGS, OS/PFS and safety; serial blood, peritoneal fluid and tissue T/NK/Treg profiling.	Tests feasibility and pharmacodynamics of local cytokine plus systemic ICI; small non-randomised design cannot prove efficacy or synergy.
HISTORIA; prospective multicentre single-arm; NCT06427252 (43)	GCPM eligible for conversion strategy.	Paclitaxel HIPEC followed by tislelizumab + SOX.	Conversion/R0 resection, response, survival, safety and QoL.	Tests a multimodal package; cannot isolate HIPEC or PD-1 contribution.
IP mesothelin CAR T-cell phase I; NCT06623396 (36)	Mesothelin-positive gastro-oesophageal adenocarcinoma with peritoneal carcinomatosis.	Regional mesothelin-directed CAR T cells with PD-1 dominant-negative receptor design.	Safety, dose finding, cellular kinetics and early antitumour activity.	Establishes regional cellular-therapy feasibility; not comparative and includes broader gastro-oesophageal disease.

CAR, chimeric antigen receptor; CRS, cytoreductive surgery; CY+, tumour-positive peritoneal cytology; EORTC, European Organisation for Research and Treatment of Cancer; FOLFOX, fluorouracil, leucovorin and oxaliplatin; GEJ, gastro-oesophageal junction; GCPM, gastric cancer peritoneal metastases; HIPEC, hyperthermic intraperitoneal chemotherapy; ICI, immune checkpoint inhibitor; IP, intraperitoneal; IV, intravenous; MSS/pMMR, microsatellite stable/mismatch-repair proficient; OS, overall survival; PCI, Peritoneal Cancer Index; PFS, progression-free survival; PIPAC, pressurised intraperitoneal aerosol chemotherapy; PM, peritoneal metastases; PRGS, peritoneal regression grading score; QoL, quality of life; SOX, S-1 and oxaliplatin; Treg, regulatory T cell.

Most are early-phase or single-arm. They can establish safety, delivery, cellular kinetics and pharmacodynamic change, but cannot determine the incremental benefit of each component. Progression through development should therefore require more than feasibility: a reproducible change in the peritoneal immune state, concordant improvement in cytology, ascites or PCI, and a clinical signal sufficient to justify a component-specific comparator.

### Separate regional treatment from operative consolidation

Future trials should distinguish two causal questions. The first is whether an intraperitoneal chemotherapy, cytokine, antibody or cellular product adds to an otherwise identical systemic backbone. The second is whether operative consolidation adds benefit amongst the minority who have sustained systemic control, cytological clearance, major PCI regression, and a high probability of CC-0/R0 resection. Immune measurements should initially be evaluated as exploratory predictors rather than unvalidated prerequisites for surgical eligibility. Randomising an operative package before deep response, then analysing only patients who reach surgery, confuses treatment effect with responder biology.

If HIPEC is included, the protocol must state whether the estimand is the entire operative package or the incremental contribution of HIPEC after complete cytoreduction. The drug, dose, carrier, temperature, duration and technique should be standardised. An immune substudy should also distinguish transient perioperative inflammation from durable restoration of antitumour immunity.

A proposed component-based pathway is shown in **Figure 1**. Its purpose is not to prescribe a single universal trial but to preserve causal clarity: the regional treatment is randomised first, response is measured directly within the cavity, and surgery is tested amongst patients meeting prespecified response criteria.

**Figure 1:**
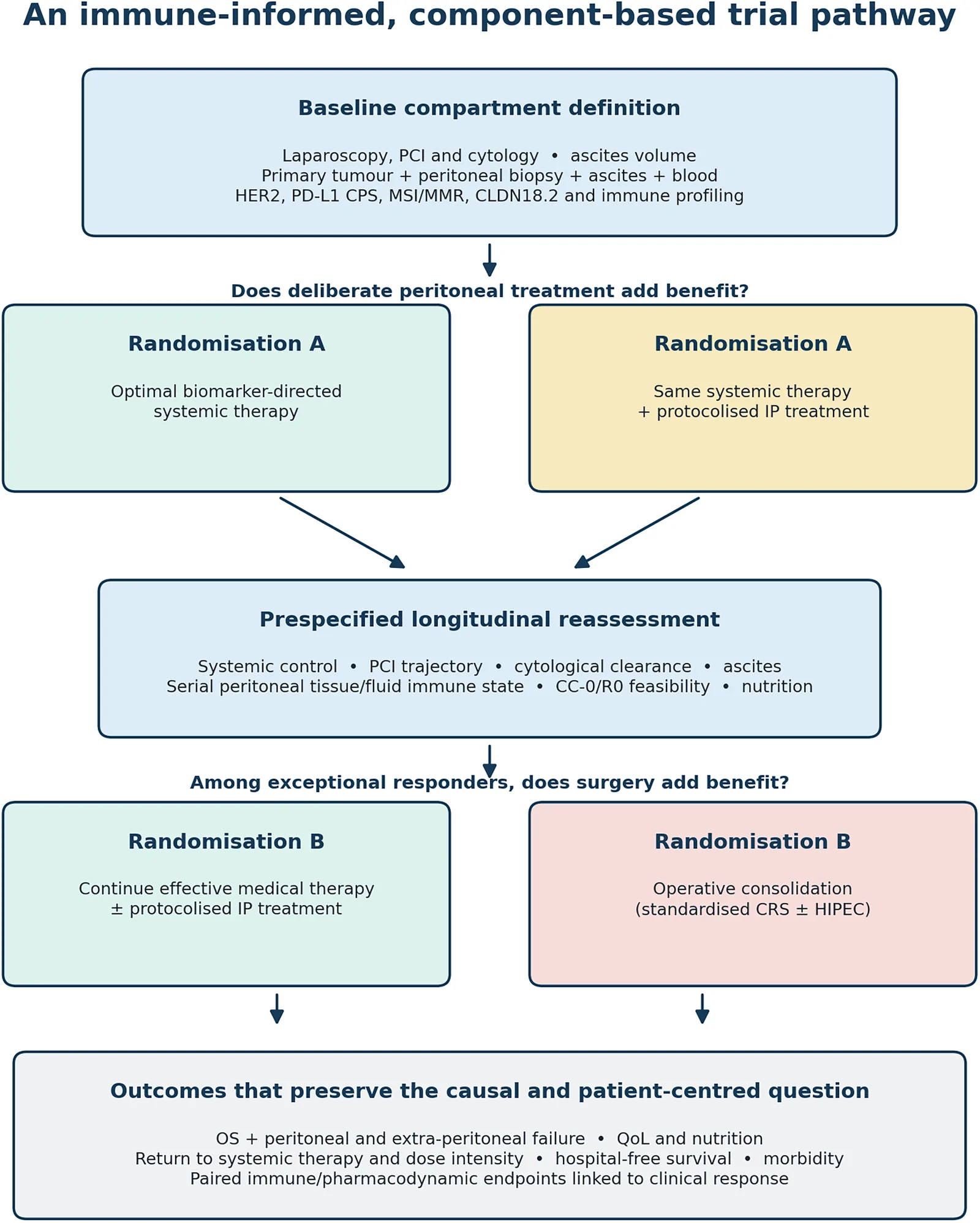
An immune-informed, component-based response-adapted pathway for future trials. The first randomisation isolates the contribution of repeated intraperitoneal treatment. Serial peritoneal and immune reassessment then defines a small exceptional-responder population for a second randomised question testing operative consolidation. Immune measurements are exploratory and are not validated eligibility criteria for surgery. CC-0, no visible residual peritoneal disease; CPS, combined positive score; CRS, cytoreductive surgery; HIPEC, hyperthermic intraperitoneal chemotherapy; IP, intraperitoneal; MMR, mismatch-repair; MSI, microsatellite instability; OS, overall survival; PCI, Peritoneal Cancer Index; QoL, quality of life; R0, microscopically margin-negative gastric resection.

### Serial immune mapping of the peritoneal compartment

At baseline and each clinically scheduled peritoneal assessment, blood, ascites or lavage and spatially annotated peritoneal biopsies should be collected. Prespecified analyses should measure lymphocyte abundance, clonality and exhaustion; natural-killer-cell representation; macrophage and myeloid states; tumour PD-L1 and NECTIN2; VEGF, TGF-β and complement pathways; and cellular-therapy expansion or persistence where relevant. Sampling location, treatment exposure and processing method must be reported.

Immune change should be linked to cytology, PCI, ascites volume, histological regression and the compartment of first progression. A rise in intratumoural CD8 cells is not sufficient if cytology remains positive or bowel function deteriorates; conversely, cytological clearance without RECIST shrinkage may represent meaningful compartment control. This linkage is what converts serial access from descriptive translational science into a mechanism-informed clinical endpoint.

### Opportunity cost and patient-centred endpoints

A locoregional intervention in metastatic disease has an opportunity cost: time without effective systemic treatment, nutritional loss, hospitalisation, deconditioning and the risk that complications permanently remove further treatment options. This cost is particularly relevant to immune therapy, where durable systemic control may be forfeited during recovery. Thirty- and 90-day morbidity alone cannot capture it.

Trials should report time from last preoperative dose to surgery, time to first postoperative systemic treatment, the proportion restarting by prespecified intervals, regimen and relative dose intensity after restart, reasons for non-resumption and subsequent treatment after progression. Hospital-free days and days alive outside hospital should accompany OS and peritoneal-control endpoints.

Patient-reported outcomes should include global health, abdominal symptoms, early satiety, pain, bowel function, neuropathy and treatment burden. Nutritional endpoints should include weight trajectory, sarcopenia, oral intake and parenteral-nutrition dependence. For PIPAC and NIPS, failed repeat access, adhesions and port complications are treatment-limiting outcomes; for CRS–HIPEC, anastomotic leak, reoperation, intensive-care stay and functional recovery are central to the net clinical effect.

### Reporting standards for an interpretable field

Comparability will remain poor unless trials report a common peritoneal dataset. Baseline reporting should include synchronous versus metachronous PM; isolated versus multi-site disease; cytology; ascites volume; total PCI and regional distribution; histological subtype; HER2, PD-L1 CPS, MSI/MMR and CLDN18.2; prior treatment; nutritional status; and performance status. At each reassessment, trials should report the same peritoneal variables and distinguish imaging, laparoscopic, cytological and pathological response.

For surgery, the report should include intended and delivered procedures, CC score, R0 status, organ resections, operating time, blood loss, intensive-care and hospital stay, morbidity, mortality and treatment resumption. Outcomes should be presented from randomisation, not only amongst treated or operated subsets. When post-randomisation subgroups are shown, landmark methods and explicit acknowledgement of immortal-time and response-selection bias are essential.

## Discussion

The clinical and translational evidence addresses different levels of inference. Patient profiling describes an immunosuppressive peritoneal environment (9–13, 18), whereas retrospective ascites cohorts identify adverse outcomes without establishing a treatment-by-ascites interaction (14, 15). Accordingly, malignant ascites is a clinically important stratification variable, but it is not yet a validated reason to withhold checkpoint inhibition. Experimental mechanisms should inform prospective hypotheses rather than substitute for predictive clinical evidence.

Regional studies also differ in what they establish. DRAGON-01 supports sustained intraperitoneal paclitaxel within its tested treatment setting (45). PIANO supplies serial evidence of local immune change (19), whilst IPLUS and the HIPEC–camrelizumab study primarily support feasibility of combined treatment (41, 42). These findings should not be pooled conceptually as proof of immune synergy. The operative trials further emphasise that any potential gain must be weighed against complications and reduced access to systemic therapy (48–52).

The practical research priority is therefore to test regional treatment on a contemporary systemic backbone, relate direct peritoneal response to serial immune measurements, and evaluate operative consolidation separately. This sequence would allow a favourable biological observation to be tested against outcomes that matter to patients, including durable control, nutritional recovery and time outside hospital.

## Conclusion

GCPM should be assessed as an interacting anatomical and immune compartment. Current evidence supports direct measurement of peritoneal disease and investigation of rational immune combinations, but does not establish universal checkpoint resistance or synergistic benefit from regional treatment.

Future trials should distinguish the added effect of intraperitoneal therapy from that of subsequent surgery, incorporate matched longitudinal blood, fluid and tissue sampling, and assess clinical benefit alongside treatment continuity, nutrition, quality of life and hospital-free survival.
